# Early antibody response and clinical outcome in experimental canine leishmaniasis

**DOI:** 10.1038/s41598-019-55087-w

**Published:** 2019-12-09

**Authors:** Ana Isabel Olías-Molero, María J. Corral, María Dolores Jiménez-Antón, José Mª Alunda

**Affiliations:** 1 Department of Animal Health, Faculty of Veterinary Medicine, Group ICPVet, Complutense University of Madrid (UCM), Avda. Puerta de Hierro s/n, 28040 Madrid, Spain; 2 Research Institute Hospital 12 de Octubre, Avda. de Córdoba s/n, 28041 Madrid, Spain

**Keywords:** Diagnostic markers, Parasitic infection

## Abstract

Infected dogs are the main reservoir of zoonotic visceral leishmaniasis, a widespread parasitic disease caused by *Leishmania infantum*. Therefore, the control of canine infections is required to reduce the incidence of human cases. Disease outcome in dogs depends on the fine balance between parasite virulence and efficacy of the immune system. Thus, knowledge of early response could yield relevant information for diagnosis and follow-up. In our study, 20 Beagle dogs were intravenously infected with 10^8^ amastigotes of a fresh isolate of *L. infantum* and monitored along 16 weeks post inoculation. Specific antibody response and clinical evolution of infected animals were highly variable. Immunofluorescence antibody test (IFAT) and enzyme linked immunosorbent assay (ELISA) were useful to assess infection status, although only ELISA with promastigote-coated plates and, particularly, western blotting (WB) allowed an early diagnosis. Prominent antigens were identified by mass peptide fingerprinting. Chaperonin HSP60, 32 and 30 KDa antigens were recognized by all dogs on week 10 post infection. This suggests that these antigens may be valuable for early diagnosis. Advanced infection showed, in addition, reactivity to HSP83 and HSP70. Disease outcome did not show a clear relationship with ELISA or IFAT titers. Correlation between the clinical status and the combined reactivity to some antigens sustains their use for diagnosis and follow-up.

## Introduction

Visceral leishmaniasis by *Leishmania infantum* (=*L. chagasi*) is a fatal unless treated vector-borne zoonotic disease prevalent in areas of South America, southern Europe and Asia^1–3^. Geographical distribution of the disease has increased and infections have been notified in northern regions (Germany, USA and Canada)^4,5^. Progression of the human infection is linked to non-effective immune system and therefore, the disease is more frequent in children, elderly, and in individuals with impaired response due to autoimmune diseases, intercurrent infections including HIV-infected patients, and iatrogenic suppression (recipients of solid organs transplants)^6–8^. Infected dogs are considered the main reservoir for zoonotic visceral leishmaniasis^2,9,10^ despite the potential role played by other hosts^11–14^. Canine leishmaniasis is a first order veterinary pathology found in dogs of all ages, breeds and conditions causing a systemic disease with both cutaneous and visceral involvement^15^. In endemic regions canine infections are very frequent with prevalence ranging from 5–8% to over 30%^16^ depending on the analytical technique employed and the sampling methodology.

There is no immune prophylaxis for human visceral leishmaniasis; new target populations, such as intravenous drug users, have been identified^17^; and chemotherapy has important shortcomings^1,2^. Given the complex epidemiology of the disease, integrated control must necessarily include the reduction of canine infections by *L. infantum* and, therefore, their transmission potential. However, effective control is hampered by the limitations of canine anti-*Leishmania* vaccines^18^, insufficient efficacy of chemotherapy against canine leishmaniasis^19,20^ and debatable impact of the environmental control, reduction of transmission by sand flies and dog culling^21–23^. Probably, success will require combination of different strategies and an early diagnosis system would be an important tool to identify newly infected (and relapsed) animals with the final aim of reducing the number of animals acting as infectious sources^24^. It is considered that dogs clinically affected by leishmaniasis have insufficient Th1 (IFN-γ) and enhanced Treg (IL-10) activity^25^, this scenario leading to overproduction of immunoglobulins, a key characteristic of canine leishmaniasis. Therefore, a variety of techniques (IFAT, ELISA, western blotting –WB-), with different levels of sensitivity and specificity^26–28^, have been used to diagnose canine infections. Moreover, several recombinant antigens have been tested^29–32^.

The main drawback of most cross-sectional studies relates to the potential cross-reactivity with other pathogens frequently coinfecting dogs (*Babesia*, *Ehrlichia*, *Neospora*, *Toxoplasma*)^33,34^ and the lack of information on the actual time elapsed after inoculation. These limitations could be overcome by analyzing experimentally infected animals to determine early infection markers and, potentially, the value of the reactivity pattern of WB for clinical follow-up. Published longitudinal studies with experimentally infected dogs by *L. infantum* are hardly comparable due to the different infective doses and via of inoculation, age and breed of experimental dogs^35^. Most of them involved low numbers of animals^36–40^ or the experiments did not include WB determinations^26,41–43^.

In the course of an unrelated project, involving a considerable number of dogs experimentally infected with *L. infantum*, serial serum samples were obtained along infection. Humoral response of the animals was determined (IFAT, ELISA, WB) with the aim of identifying early infection markers, immune detection patterns, correlation between the diagnostic techniques and their relationship to the clinical status of the animals.

## Results

### Serum antibody response estimated by IFAT and ELISA

**Figure 1:**
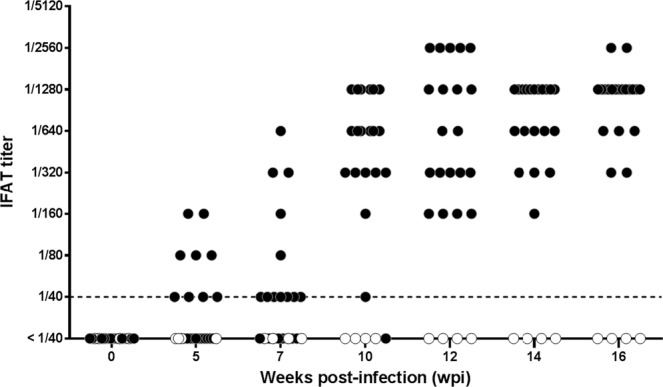
Serum anti-*Leishmania* response of experimentally infected Beagle dogs along the experiment determined by IFAT. Solid circles: individual IFAT values of infected dogs (n = 20); empty circles: uninfected control animals (n = 4). Dashed line: cut-off titer.

**Figure 2:**
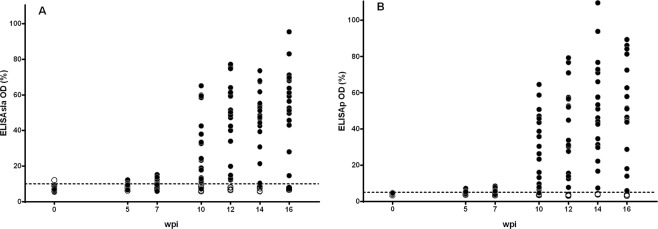
Individual response estimated by ELISA of Beagle dogs infected with *L. infantum* (solid circles) (n = 20) and uninfected control animals (empty circles) (n = 4) along the experiment. Y axis values: % of optical density (OD) from positive control animals. Dashed line: cut-off value. (**A**) ELISA with soluble leishmanial antigen (ELISAsla). (**B**) ELISA with promastigote-coated plates (ELISAp). Weeks post infection: wpi.

Female Beagle dogs (10–11 months old) were inoculated with 10^8^ amastigotes of *L. infantum* freshly obtained from a naturally infected dog (n = 20) or kept as uninfected control animals (n = 4). Dogs were housed under controlled conditions precluding undesired arthropod-borne infections, daily observed and subjected to periodical clinical explorations and biochemical and immunological evaluations along 16 weeks post inoculation. Uninfected control dogs did not show any specific antibody response along the experiment. Inoculation of dogs with *L. infantum* elicited a time-dependent increase of IFAT titers along the infection and 5 weeks post infection (wpi) five animals were over the threshold titer (≥1/80) (Fig. 1); five weeks later (week 10 pi) the majority of the inoculated dogs (18 out of 20) were IFAT+ and 12 wpi all animals showed titers ≥1/160. Immune response was heterogeneous and on week 16 pi IFAT titers ranged from 1/320 to 1/2560. Specific response estimated by ELISA with soluble *Leishmania* antigen (ELISAsla) (Fig. 2A) and ELISA using promastigotes as antigen (ELISAp) (Fig. 2B) showed a comparable pattern, all infected animals being positive by week 12. Despite individual variation, there was a strong correlation between both ELISA tests (r = 0.9376, *P* < 0.0001). IFAT values did correlate with ELISAp (r = 0.8632; *P* < 0.0001) and ELISAsla (r = 0.8487; *P* < 0.0001). ELISAp allowed an earlier diagnosis of *L. infantum* infection since 5 wpi the technique detected eight positive animals whereas only five animals were positive by IFAT and ELISAsla. Seven wpi the advantage of ELISAp for early diagnosis was more evident since 13 dogs were positive *versus* 5 animals by IFAT and 7 by ELISAsla. Accordingly, there was a pi time variation of Cohen’s Kappa coefficient value (Table 1). Thus, the agreement between ELISA and IFAT after 10 weeks was good (κ = 0.64) but in the first sampling (5 wpi) it ranged from poor to moderate (ELISAsla/IFAT, κ = 0.20; ELISAp/IFAT, κ = 0.44).

**Table 1:** Agreement (Cohen’s κ value) between diagnostic techniques along experimental infection of Beagle dogs with *Leishmania infantum*.

	IFAT			ELISAsla			ELISAp		
	5wpi	7wpi	10wpi	5wpi	7wpi	10wpi	5wpi	7wpi	10wpi
IFAT	—	—	—	0.20	0.29	**0.64**	0.44	0.30	**0.64**
ELISAsla	0.20	0.29	**0.64**	—	—	—	0.22	0.27	**1.00**
ELISAp	0.44	0.30	**0.64**	0.22	0.27	**1.00**	—	—	—

In bold: substantial (0.61–0.80) and almost total agreement (0.81–1.00). ELISAsla: ELISA with soluble leishmanial antigen; ELISAp: ELISA with promastigote-coated microplates. wpi: weeks post infection.

### Longitudinal study of western blotting pattern

**Figure 3:**
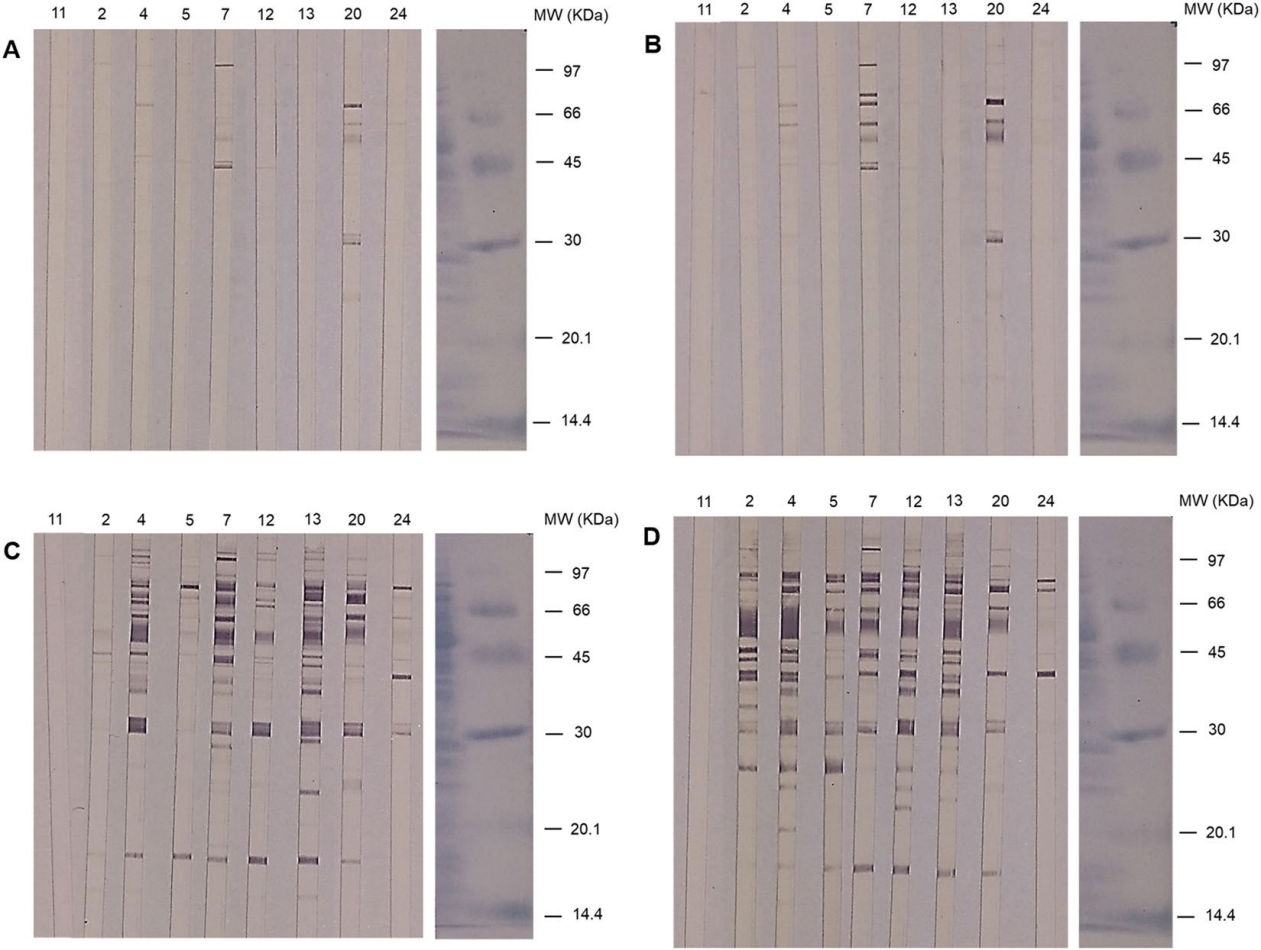
Western blot analysis of SLA fractionated by electrophoresis under denaturing and reducing conditions (SDS-PAGE) probed with individual dog sera: 5 weeks (**A**), 7 weeks (**B**), 10 weeks (**C**) and 16 weeks post infection (Fig. 4D). Numbers on the strips correspond to the identification of experimental dogs. #11. Uninfected control animal. MW: molecular weight markers in KDa. Strips were cut from the membrane, developed and mounted. Incubation of strips and development conditions were standardized. Strips on the right correspond to the control of protein transfer and MW markers, stained with Amido Black.

A selection of dogs, representing the observed range of clinical presentations, was analyzed to determine the antigen recognition pattern at different post inoculation times (5, 7, 10 and 16 wpi) (Fig. 3). There was a notable individual variation, both in intensity of reactivity and immunodominant antigens recognized, despite the identical infective dose administered and the close genetic background of dogs. Sera from infected dogs showed extensive reactivity with antigens of MW *ca*. 93, 87, 85, 77, 72, 70, 66, 56, 50, 48, 46, 44, 41.5, 40, 38, 35, 32, 30, 28, 25.5, 23.5, 23, 21.5, 19.5, 17 and 15 KDa. Faint reactivities were found when testing the sera of the uninfected control animals, mainly on ~77 Da and >97 KDa (Fig. 3, Supporting Information Fig. 1).

Analysis of WB with ImageJ software allowed the quantitation (expressed as DU or density units) of the total reactivity as well as the time-course recognition of individual antigens along the infection progress (Supporting Information Figs. 2 and 3). WB was very sensitive for early detection of canine leishmaniasis since on week 5 pi six dogs reacted with SLA (Fig. 3A; Supporting Information Fig. 3,A). However, individual recognition was highly variable and the most prominent response was found in dogs #20 and #7. Two weeks later (7 wpi) results were similar with a more complex pattern and higher intensity of reaction in dog #20 (9,292.9 DU) (Fig. 3B; Supporting Information Fig. 3,B). By week 10 pi (Fig. 3C; Supporting Information Fig. 3,C) a progressive increase of reactivity was observed in all animals, particularly the early responder dogs (e.g. #7: 23,920.7 DU). Some dogs showing scarce reactions in the previous samplings (#5, #12, #13, #24) displayed extensive recognition of SLA at this time. WB performed with sera from 16 wpi (Fig. 3D; Supporting Information Fig. 3,D) were comparable to those obtained with sera of dogs with natural chronic infections (not shown). From week 10 pi onwards all infected dogs specifically and significantly reacted with some regions (30, 32 and, especially, ~56 KDa), and by week 16 pi also with 41.5, 66 and 85 KDa (Table 2). Thus, these antigens could be employed, if not shared with other dogs’ pathogens, for diagnosis and follow-up. Despite the limited analysis, total WB reactivity (DU) of dogs did correlate both with ELISAp (r = 0.9132; *P* < 0.0001) and ELISAsla (r = 0.805; *P* < 0.0001) (r > 0.84; *P* < 0.0001) (Supporting Information Fig. 4).

**Table 2:** Statistical differences (*P* value in Mann-Whitney U test) between the reactivity (Density Units, DU) of sera of Beagle dogs experimentally infected with *Leishmania infantum* and uninfected control animals, with some selected antigens of *L. infantum*, along the experimental period*.

	*P* value			
	5 wpi	7 wpi	10 wpi	16 wpi
85 KDa	Non-significant	Non-significant	0.0182	0.0040
56–66 KDa	Non-significant	Non-significant	0.0040	0.0040
32 KDa	Non-significant	Non-significant	0.0040	0.0040
30 KDa	Non-significant	Non-significant	0.0040	0.0040
85+ 56− 66+ 32+ 30 KDa	Non-significant	Non-significant	0.0040	0.0040

*Level of significance, *P* < 0.05.

**Figure 4:**
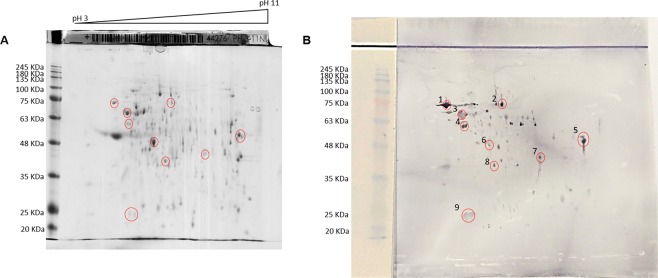
(**A**) 2D electrophoretic separation of soluble *Leishmania* antigens (SLA). (**B**) Western blot of SLA with serum (1/50) from a dog with a chronic *L. infantum* infection. Two 2D gels were run in parallel: the first one (**A**) was stained with Coomassie blue; the second one was transferred to a PVDF membrane for Western blot (**B**). The strip on the left was used as transfer control of SLA and markers. MW: molecular weight markers in KDa; pH 3–11: pH gradient. Circles: selected spots for peptide identification by mass spectrometry and finger printing.

The 2D electrophoretic separation of *L. infantum* SLA (Fig. 4A) and the recognition by serum from a chronically infected animal (Fig. 4B) is shown in Fig. 4. Nine spots, corresponding to the immunodominant antigens in WB, were selected for identification by mass spectrometry. Isolated proteins corresponded to 1: Heat shock protein 83 (HSP83) (Mr 73,939), 2: putative methylmalonyl-CoA mutase (Mr 79,948), 3: HSP70 (Mr 69,981), 4: Chaperonin HSP60 (Mr 59,831), 5: Elongation α factor (Mr 44,191), 6: enolase (Mr 47,095), 7: putative HSP DNA.J (Mr 44,994), 8: putative arginine kinase (AK) (Mr 42,363), 9: putative glutathione peroxidase-like (Mr 19,587) (Supporting Information Table 1).

### Relationship between clinical course and antibody response

Clinical status of the dogs, according to the clinical scoring (CS) of the animals after 16 weeks of *Leishmania* infection, did not correlate with the IFAT titers (Supporting Information Fig. 5). However, clinical status correlated with ELISA (ELISAsla/CS: r = 0.6546; *P* = 0.0017; ELISAp/CS: r = 0.6614; *P* = 0.0015) (Supporting Information Fig. 6). CS correlated with the total WB reactivity of infected dogs (r = 0.879; *P* = 0.0091) (Supporting Information Fig. 7) although the relationship was not linear: dogs with the highest CS on week 16 (#4: 19, #13: 19) had very different DU (>36,000 DU *vs*. 25,465.55 DU, respectively) (Supporting Information Fig. 3,D). Analysis of reactivity to immunodominant antigens showed that 30 KDa and 32 KDa slightly correlated with CS (r = 0.763; *P* = 0.0389 and r = 0.80; *P* = 0.025, respectively). However, combined reactivity of some antigens displayed a higher correlation, particularly when DU of 85 + 66–56 + 32 + 30 KDa antigens on week 16 pi was considered (r = 0.9092; *P* = 0.005) (Supporting Information Fig. 8).

## Discussion

All inoculated dogs were infected and developed clinical signs and lesions compatible with leishmaniasis as well as a strong anti-*Leishmania* specific antibody response. This supports the model and experimental design used, including *L. infantum* strain, infective dose, leishmanial stage and via of inoculation. Individual variability of the animals along the infection, both in the clinical course and the immune response, is the rule in experimental canine leishmaniasis^26,40–45^ despite the close genetic background of experimental Beagle dogs.

IFAT is considered the golden standard technique for diagnosis of canine leishmaniasis in clinical practice^9,46^ and its sensitivity and specificity, compared to ELISA, for diagnosis and follow-up purposes has been tested^26,39,41,46,47^. As regards the diagnostic value of different formats of ELISA including recombinant antigens (rK39, rK28)^26,45,48^ and synthetic peptides^30^, it has been determined in cross-sectional and longitudinal studies. Results have shown that, in general, ELISA has higher sensitivity than IFAT^47^. Both in-house IFAT and ELISA tests (ELISAp and ELISAsla) were, under our conditions, adequate diagnostic procedures after 12 weeks of infection as reported previously^41^. However, follow-up of inoculated dogs showed that early phases of the infection were only detected by ELISAp, and this method was on average >2.5 fold more sensitive than standard ELISA and IFAT (7 wpi). ELISAp allowed, in our case, an earlier diagnosis than those reported with standard ELISA and rK39 immunochromatographic test in experimentally infected dogs (90–120 days pi)^26,42,45^. There was a good agreement between IFAT titration and ELISA results (κ > 0.6) at 10 wpi, but not earlier, and ELISAsla and ELISAp produced comparable results at that time (κ = 1.0). Moreover, IFAT test is time consuming and requires skilled personnel and expensive equipment. Since earlier diagnosis is an advantage for the clinical management of dog leishmaniasis, ELISAp could be a convenient diagnostic choice compared to standard ELISA and IFAT to detect acute *L. infantum* infections in dogs.

WB is a highly sensitive technique proposed for diagnosis and as prognostic marker of canine leishmaniasis^49–53^. In the present study, sensitivity of WB was superior (100% after 10 weeks of infection) to that achieved with the other techniques tested (IFAT, ELISAp, ELISAsla), this confirming previous results^36–38^. In addition, WB was more precocious (10 wpi) than some rtQ-PCR (17 wpi)^43^.

There is no global consensus on the WB recognition pattern by sera of *L. infantum* infected dogs. WB banding found in experimental infections^36–39,54^ are simpler than those found in naturally infected dogs^39,51–53^ although, in these cases, the possibility of coinfections with other antigen-sharing pathogens could not be ruled out. WB reactivities from our study, against immunodominant antigens of 85, 66, 56, 41.5, 32 and 30 KDa, were comparable to those obtained in experimentally infected dogs of the same breed^36^ and in mixed breed animals^37^. Besides the diagnostic value of WB in established *L. infantum* infections, follow-up allowed the determination of time-related antigen recognition. Under our conditions, there was considerable variability among animals, and reactivity to the 56 KDa antigen and, less clearly, to the 32 and 30 KDa antigens was observed in all inoculated dogs only after 10 weeks of infection. It is possible that the apparent delay in the development of specific antibodies found, compared to previous reports^36,38^, would be related to the different experimental design, parasite strain and infective dose, individual immune response and methodology employed. Mass spectrometry allowed the identification of *L. infantum* antigens along the infection. Three of the immunodominant antigens were heat-shock proteins (HSP83, HSP70 and chaperonin HSP60), of poorly known biological functions^55,56^ although significant immunogens in *Leishmania* infections^57^. HSP83 and HSP70 are recognized in rodent models and their simultaneous reactivity is considered a marker of visceral leishmaniasis^58^. These HSPs have been proposed as diagnostic antigens in dog leishmaniasis although HSP70 apparently cross reacts with *Trypanosoma cruzi*^59,60^ this eventually leading to unspecific results in co-endemic areas. Diagnostic value of chaperonin HSP60 is less known although it has been reported to react with sera of dogs with subclinical natural infection^53^. Since, in our case, by week 10 pi *L. infantum* HSP60, 32 KDa and 30 KDa antigens were recognized by all inoculated dogs, their combination would allow an early diagnosis of canine infection. WB is not routinely employed in many diagnostic laboratories, but these antigens could be used in a dot-ELISA format, as suggested for human leishmaniasis^61^. Alternatively, epitope mapping could allow the construction of recombinant chimeric proteins. This approach has been followed with other proteins (PQ10, PQ20) in cross-sectional and a limited longitudinal study although the multiepitope-based ELISA required 4–5 months of infection to be positive^30,31^. Our results, and the present availability of recombinant *Leishmania* HSPs^56,62^ and serial serum samples of experimentally infected dogs, could be used to confirm their diagnostic value under field conditions.

IFAT titration is frequently used in veterinary practice as a reliable method for monitoring the clinical evolution of *L. infantum* infected dogs, including their response after chemotherapy. Our results showed that, contrary to this assumption, IFAT did not show any significant correlation with the clinical status (CS) of the animals; therefore its value for disease follow-up and post treatment monitoring^44,63^ should be reconsidered. However, it is worth indicating that results of IFAT are not lineal and serum titration was stopped at 1/2560 dilution whereas in ELISA actual OD values were considered. ELISA correlated better with the clinical status of animals than IFAT, although correlation was moderate. (r *ca*. 0.65). Several reports have associated WB patterns (IgG, IgG_1_, IgG_2_) to the clinical status of naturally and experimentally infected dogs^36,38,40,51,52,64^ and some antigens have been suggested as prognostic markers. In our study, combined reactivity to immunodominant antigens (HSP83, HSP70, HSP60, 32 KDa and 30 KDa) correlated with the clinical outcome. This suggests their potential value for both diagnosis and clinical follow-up and is consistent with the hyperglobulinemia found in dog leishmaniasis. Further research with accurate determination of immunoglobulin subclasses of dogs^65^ along the *L. infantum* infection course would clarify their role in the disease outcome. Whether these findings in experimental canine leishmaniasis, with a pure dog breed and intensively monitored animals, are also present in natural infections of different dog breeds, ages and management conditions needs further research under field conditions.

## Material and Methods

### Leishmania infantum strain

Inoculum was a fresh isolate of *L. infantum* obtained from the spleen of a naturally infected dog clinically and serologically diagnosed (Órgiva, Granada, Spain). After euthanasia, spleen was aseptically removed, and transported to our facilities under refrigeration. The organ was cut into small pieces (*ca*. 5 mm^3^), and homogenized in a glass-in-glass tissue grinder (5 mL phosphate buffered saline, PBS). Suspensions were centrifuged twice (50 × *g*, 10 min; 1100 × *g*, 10 min, 4 °C). Cell pellets were treated for 30 seconds with cell lysis buffer (SDS 0.05%), resuspended in PBS and amastigotes counted in an improved Neubauer chamber. Isolation was performed ice-cooled under sterile conditions and amastigotes were kept at 4 °C and used to inoculate dogs after 24 h. The isolate was characterized using published kinetoplast primers^66^ and by a specific PCR-hybridization-ELISA with a cloned 196 bp of *L. infantum* kDNA^67^. Both analyses confirmed the isolate as *L. infantum*, provisionally labeled as MCAN/ES/2016/Granada-UCM.

### Experimental infection of dogs with *L. infantum* and follow-up

Female Beagle dogs (24 animals) were obtained from Envigo (France) when they were 4–5 months old and housed at the Faculty of Veterinary Medicine UCM (Madrid) (Animal facility Nr ES280790000091). Animal facilities were fitted with mosquito nets precluding the access of sand flies. Periodical complete physical exploration, biochemical, hematological and immunological evaluations showed physiological normality and negative IFAT test to *L. infantum*. When the animals reached 10–11 months age, 20 randomly selected animals were inoculated intravenously (cephalic vein) with 10^8^ amastigotes of *L. infantum*/animal, administered in 1 mL. Four animals were kept as uninfected control dogs. After inoculation, dogs were daily observed, and every 2 weeks, weighed and subjected to complete clinical examination by a veterinarian blinded to the experimental design. Blood samples were obtained from the cephalic vein and routine immune response test (IFAT) was carried out by an external laboratory (Lab. Barba, Madrid). Dog sera were considered positive with IFAT titer ≥1/80. Infection status of inoculated animals was assessed on week 16 pi by popliteal lymph node sampling and microscope observation of amastigotes in stained smears (May Grünwald-Giemsa). Infected animals displayed a course-related range of clinical signs and lesions characteristic of leishmaniasis including lymph node enlargement, splenomegaly, skin lesions (*e.g*. erythema, alopecia), ocular lesions (*e.g*. conjunctivitis), paleness of mucosal membranes and muscular atrophy. Clinical status of the animals was quantified with a clinical score (CS) based on Manna *et al*.^68^ and Foglia-Manzillo *et al*.^69^ including clinical signs, lesions and hematological and biochemical abnormalities (maximum 35 points) (Supporting Information Table 2).

### Antigen preparation

Promastigotes obtained by back transformation of amastigotes from the original isolate used for infection, were cultured in 175 cm^2^ culture flasks at 27 °C in RPMI 1640 modified medium (BioWhittaker) supplemented with 10% heat-inactivated (30 min at 56 °C) fetal bovine serum (Gibco), 100 U/mL penicillin plus 100 μg/mL streptomycin (BioWhittaker), 1% L-glutamine (BioWhittaker) and 1% human urine. To obtain SLA for ELISA and WB mid-log phase promastigotes were frozen at −80 °C, subjected to 5 freezing-and-thawing cycles (liquid nitrogen-water bath at 37 °C) and centrifuged at 18000 × *g* for 20 min at room temperature (RT). Supernatants were collected and protein concentration was determined with RC-DC Protein Assay (BioRad). For ELISAp, promastigotes (10^9^ cells/mL) were fixed with 0.025% formaldehyde (Panreac) in PBS for 2 h at RT, counted in improved Neubauer chamber and used to coat microtiter plates.

### ELISAsla and ELISAp conditions

Optimal assay conditions of ELISA were determined in a checkerboard manner. For ELISAsla, 96-well plates (Nunc Maxisorp, Thermo Fisher Scientific) were coated with 20 μg/mL (50 μL/well) of SLA overnight at 4 °C, blocked (PBS-2% BSA) for 1 h, 37 °C and diluted dog sera (1/400) added (50 μL/well) and incubated (2 h, 37 °C). Secondary antibodies (1/5000, 50 μL/well) (goat anti-dog IgG H + L, Bethyl Laboratories) were added and plates were incubated for 1 h at 37 °C. Color was developed with 1 mg/mL *O*-phenylenediamine (Sigma) and H_2_O_2_ (1/1000) (100 μL/well). The reaction was stopped with 50 μL 3 N H_2_SO_4_ and absorbance (OD) was read at 492 nm in an Opsys MR microplate reader (Dynex Technologies).

For ELISAp, microplates were coated overnight at 4 °C with 5 × 10^6^ promastigotes/well. Plates were blocked (1 h, 37 °C, PBS- 2% BSA). Diluted dog sera were added (1/800, 50 μL/well) and plates incubated (2 h, 37 °C). Secondary antibody incubation, color development and absorbance were as above. Determinations were performed at least in triplicate. Average OD + 2 standard deviations (SD) of preinfection dog sera was the cut off value.

### Electrophoresis (SDS-PAGE) and western blotting (WB)

#### 1D SDS-PAGE and WB

SLA was analyzed by 12.5% SDS-PAGE (150 V, 150 mA). WB was carried out following a previously described method^70^. Briefly, gels were transferred onto Immobilon P (Millipore) (150 V, 400 mA). Blocked membrane strips (2.5 mm wide) were incubated with dogs’ sera (1/50) in tubes for 3 h at 37 °C and anti-dog IgG (Bethyl Laboratories) (1/1000) was added (2 mL/tube) and incubated at 37 °C, 1 h. Color was developed with chloro-1-naftol + H_2_O_2_ at RT and reaction stopped with MilliQ water. Immune recognition in WB was analyzed by ImageJ software (https://imagej.nih.gov) to determine reactivity (density units, DU). Low molecular weight (MW) markers were from GE Healthcare.

#### 2D Electrophoresis, 2D WB and peptides identification

First dimension (50 μg SLA) was run in parallel on two 3–11 NL pH gradient 8 cm strips (GE Healthcare) in Ettan IPGphor 3 IEF System (GE Healthcare) until 5 kVh. Second dimension was run on hand cast 10% acrylamide gels (BioRad gel caster and MiniProtean II chamber) at 100 V in Laemmli buffer. One gel was stained with colloidal Coomassie blue g250, and the other transferred to a PVDF membrane (BioRad, Mini Trans-Blot cell) (150 V, 2 h at 4 °C) in Towbin buffer. WB was carried out with 1/50 diluted serum from a dog chronically infected with *L. infantum*. Peptides were selected in the stained gel by comparison with 2D WB reactivity, in-gel reduced and digested with trypsin^71^. Analysis of peptides was performed using a 4800 Plus MALDI TOF/TOF mass spectrometer analyzer (Applied Biosystems, MDS Sciex), at the Proteomics Unit of UCM, Madrid. Peptide mass fingerprint and peptide fragmentation spectra were combined and searched in MASCOT v2.3 (http://www.matrixscience.com) through Global protein Server software (Applied Biosystems) against NCBI database. Search was performed without taxonomy restriction and the following parameters: carbamidomethyl cysteine as fixed modification and oxidized methionine as variable modification; peptide mass tolerance, 80 ppm; one missed trypsin cleavage site allowed, and MS/MS fragments tolerance, 0.3 Da. In all protein identifications, the probability scores were greater than the score fixed by Mascot as significant (*P* < 0.05).

### Statistical analysis

For statistical analysis, IFAT values were transformed (≤1/40 = 1; 1/80 = 2; 1/160 = 3; 1/320 = 4; 1/640 = 5; 1/1280 = 6; ≥2560 = 7). ELISA values were expressed as percentage (%) of the OD value found for 20 pooled sera of dogs naturally infected with *L. infantum*, serologically and parasitologically confirmed, obtained from the Clinical Services of the Faculty of Veterinary Medicine UCM (OD of samples/Average OD of positive control population x 100). Agreement of diagnostic techniques was determined with Cohen’s Kappa index. Relationship between the different diagnostic techniques, as well as between CS and diagnostic techniques, were evaluated using the non-parametric Spearman correlation^72^. Differences between DU of individual antigens recognized in WB by infected and control dogs were determined with Mann-Whitney U non-parametric test. In all statistical analyses level of significance was set at *P* < 0.05. Statistical analysis and figures were done with Graphpad Prism 6.01.

### Compliance with ethical standards

Principles established by the European Commission legislation (Directive 63/2010/EU) and Spanish national transposition (Royal Decree 53/2013) on protection of animals used for scientific purposes, and 3Rs principles were followed. Experimental design and procedures were approved by the Ethical Committee (Faculty of Veterinary Medicine UCM, Madrid); the Committee for Animal Experimentation (UCM), and the Animal Health authorities from the Regional Government of Madrid (Ref. PROEX 329/15). All personnel in direct contact with the animals had official qualification for animal handling and experimentation (ECC/566/2015).

## Supplementary information

Supplementary Information
