# Supplementary material for: Early antibody response and clinical outcome in experimental canine leishmaniasis
Source: Sci Rep. 2019 Dec 9;9:18606. doi: 10.1038/s41598-019-55087-w (PMC6901516; doi:10.1038/s41598-019-55087-w)
Supplement: Supplementary file 1 — Supplementary Information [file 41598_2019_55087_MOESM1_ESM.pdf]

Manuscript SREP-19-23559-T

SUPPLEMENTARY INFORMATION

Early antibody response and clinical outcome in experimental canine leishmaniasis

Ana Isabel Olías-Molero, María J. Corral, María Dolores Jiménez-Antón, José M[a] Alunda

Department of Animal Health. Faculty of Veterinary Medicine, Complutense University of Madrid (UCM), Avda. Puerta de Hierro s/n, 28040 Madrid, Spain

Correspondence:
José M[a] Alunda
Email: jmalunda@ucm.es

Legend to figures

Supplementary Information Fig. 1: Western blot analysis of SLA fractionated by electrophoresis under denaturing and reducing conditions (SDS-PAGE) probed with individual sera of uninfected control dogs at 5, 7, 10 and 16 weeks post infection (wpi). Numbers on the strips correspond to the identification of experimental dogs. Strips were cut from the membrane, developed and mounted. Incubation of strips and development conditions were standardized. Strip on the right: control protein transfer and MW markers, stained with Amido Black. MW: molecular weight markers in KDa.

Supplementary Information Fig. 2: Representative figures of reactivity determined by WB. Figure 1A: early responder dog (dog #20). Figure 1B: late responder dog (dog #l3). Density corresponds to the output from ImageJ software. MW: Molecular weight in KDa.

Supplementary Information Fig. 3: Reactivity to individual antigens along the experimental infection of Beagle dogs 5 (Fig. 3A), 7 (Fig. 3B), 10 (Fig. 3C) and 16 (Fig. 3D) weeks post inoculation with *L.infantum*. Reactivity was determined with ImageJ software and output in density units (DU) transformed to color densities (heat map). Clinical score (CS), ELISAsla, ELISAp and IFAT results are included for comparative purposes using a color scale. Grey color cells: DU from these cells could not be differentiated. MW: molecular weight in KDa.

Supplementary Information Fig. 4: Relationship between total reactivity determined by WB and ELISA values using soluble *Leishmania* antigen (ELISA sla) (Fig. 4A) or promastigotes as antigen (ELISAp) (Fig. 4B) of dogs experimentally infected with *Leishmania infantum*. Black circles correspond to individual dogs' sera. DU: Density Units as determined by ImageJ.

Supplementary Information Fig. 5: Relationship between immunofluorescence antibody test (IFAT) titers and the clinical score (CS) of dogs experimentally infected with *L. infantum*. Black circles correspond to individual dogs on week 16 post infection.

Supplementary Information Fig. 6: Relationship between clinical score (CS) and ELISAsla (Fig. 6A) and ELISAp (Fig. 6B) of dogs experimentally infected with *L. infantum*. Black circles correspond to individual dogs on week 16 post infection.

Supplementary Information Fig. 7: Relationship between individual reactivity determined by WB and clinical score of dogs experimentally infected with *L. infantum*, 16 wpi. Black circles correspond to individual animals. DU: Density units as determined by ImageJ.

Supplementary Information Fig. 8: Relationship between the recognition of *L. infantum* antigens (85 +56-66 + 32 +30 KDa) and the clinical score of dogs experimentally infected with *L. infantum* on week 16 pi. DU: Density units as determined by Image J. Black circles correspond to individual dogs.

Tables

Supplementary Information Table 1: Alignment of identified peptides by Mass Spectrometry and finger printing in the 2D-electrophoresis (in bold) and the sequences of proteins of *Leishmania* or trypanosomatids.

Supplementary Information Table 2: Clinical scoring (CS) of Beagle dogs experimentally infected with *Leishmania infantum* considering clinical signs and lesions (20 points) and hematological and biochemical abnormalities (15 points). Maximum Score: 35 points

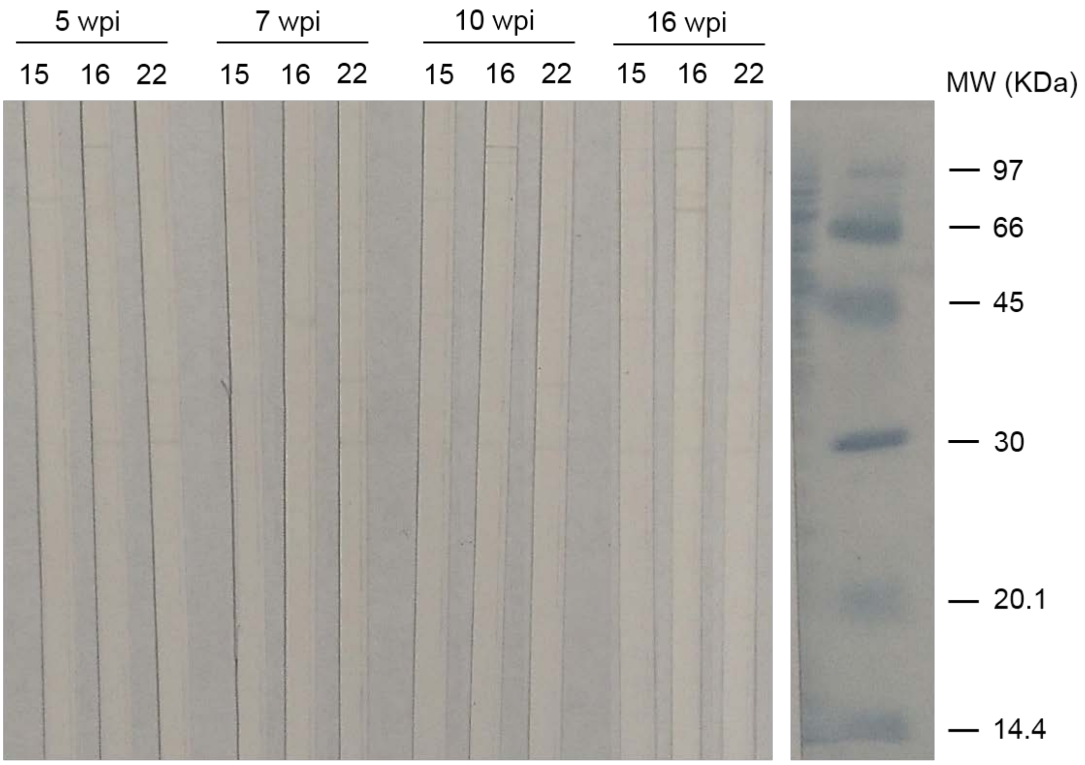
5 wpi
7 wpi
10 wpi
16 wpi
15 16 22 15 16 22 15 16 22 15 16 22
MW (KDa)
97
66
45
30
20.1
14.4

Figure 1

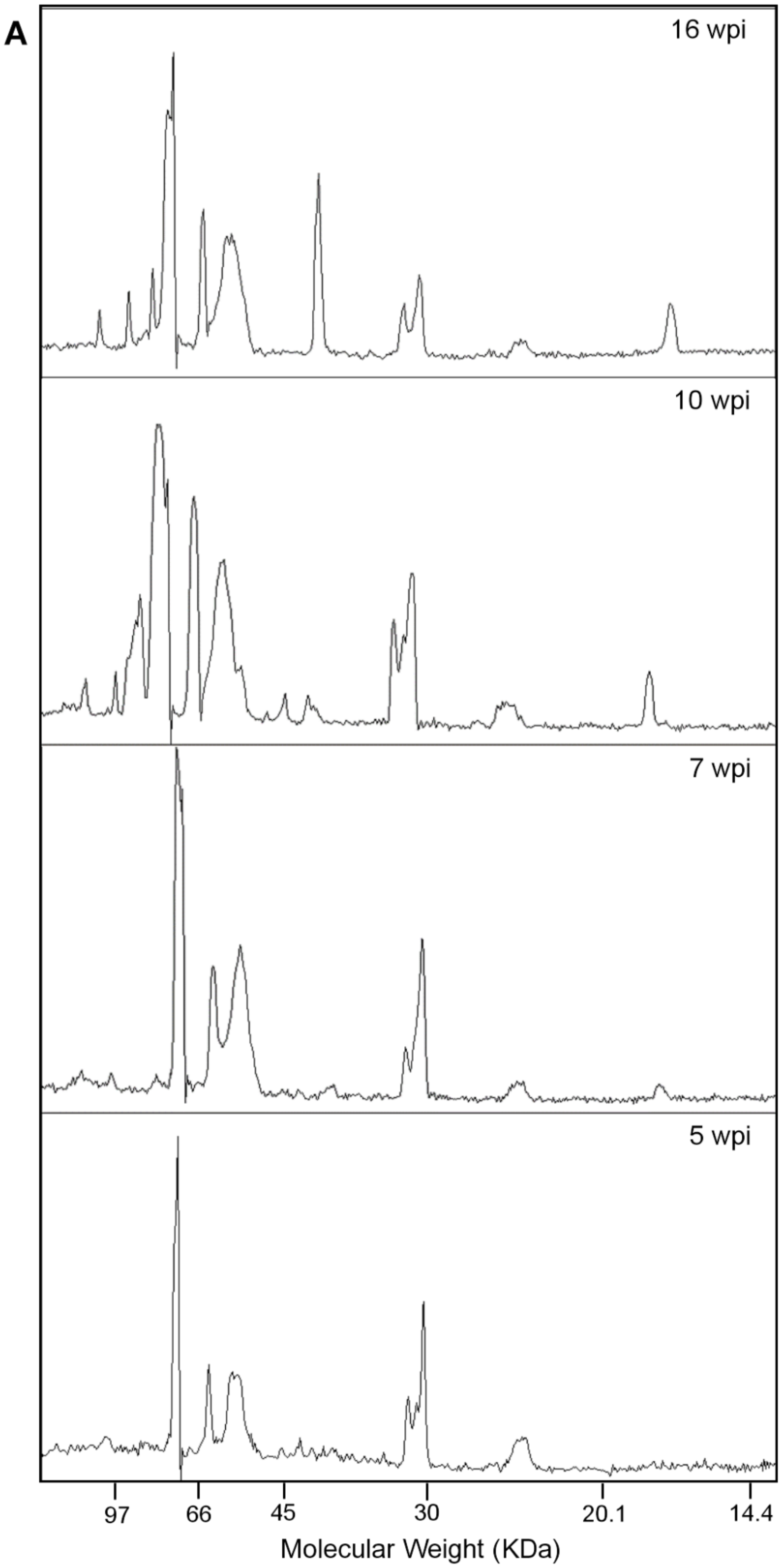
A
16 wpi
10 wpi
7 wpi
5 wpi
97
66
45
30
20.1
14.4
Molecular Weight (KDa)

SI Figure 2A

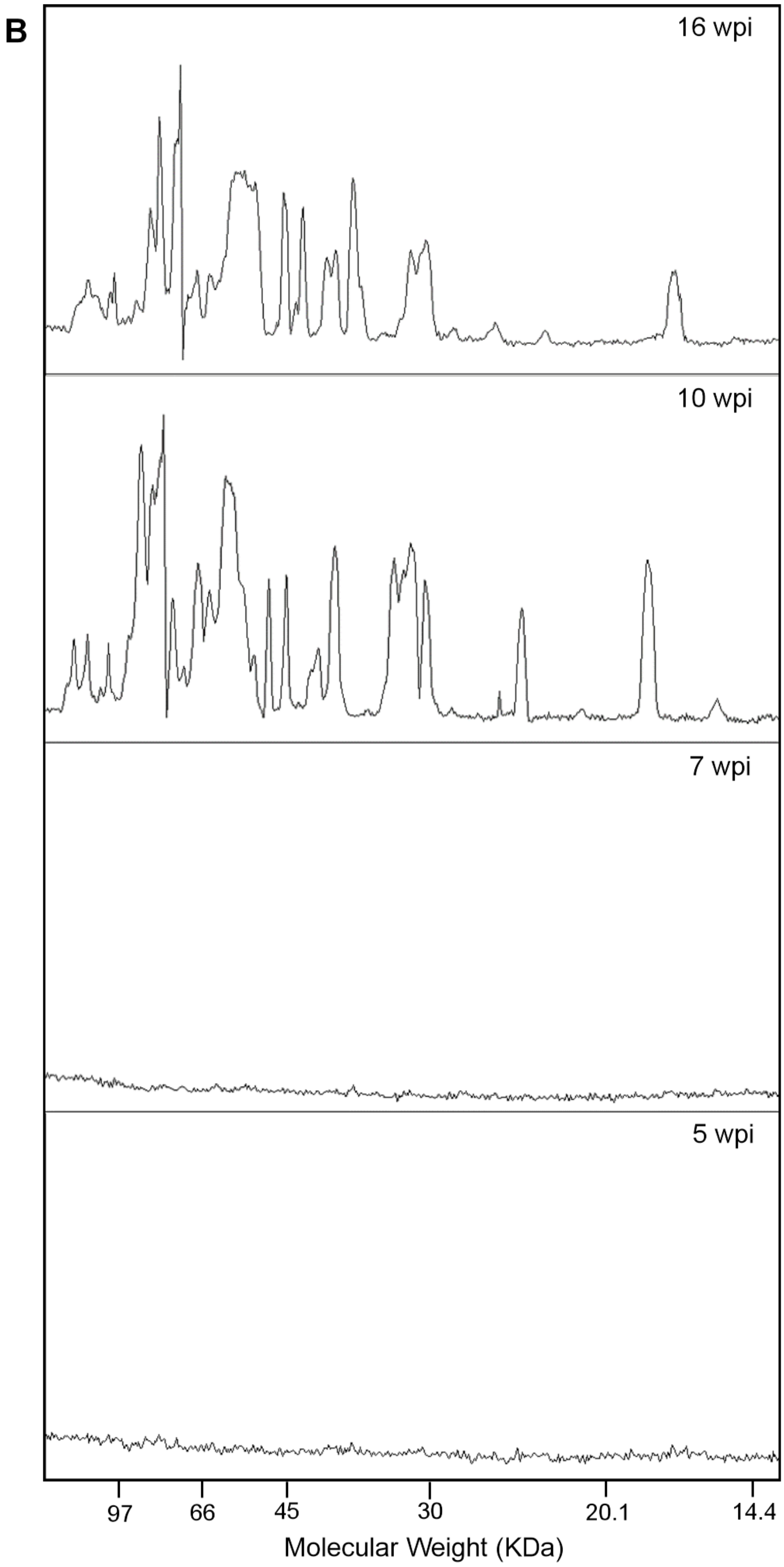
B
16 wpi
10 wpi
7 wpi
5 wpi
97
66
45
30
20.1
14.4
Molecular Weight (KDa)

SI Figure 2B

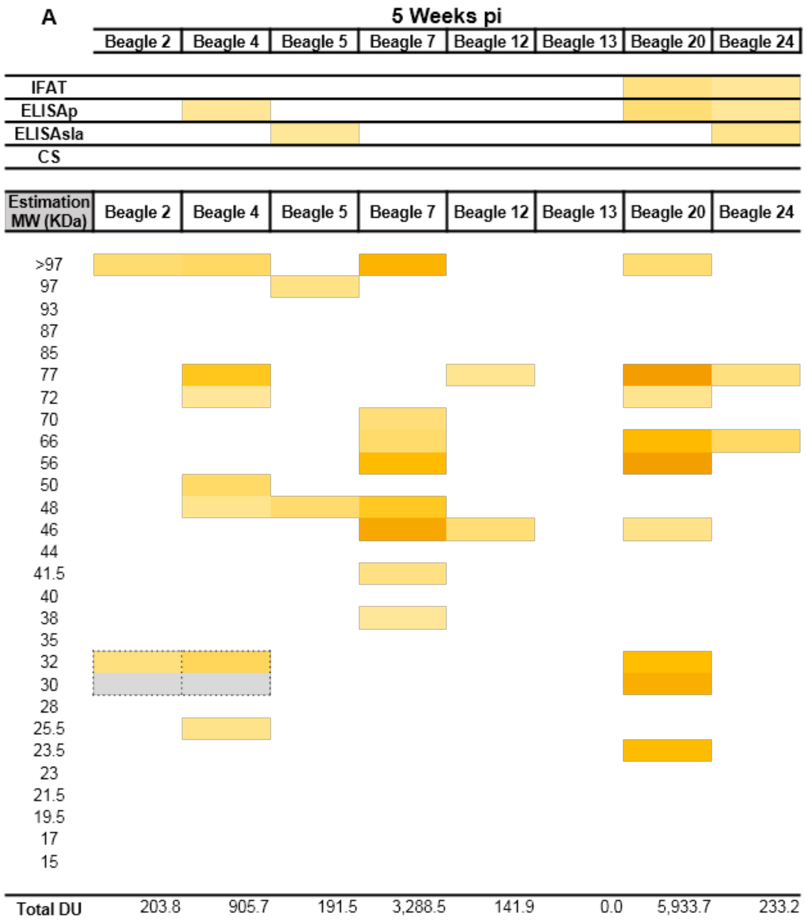
A
5 Weeks pi
Beagle 2
Beagle 4
Beagle 5
Beagle 7
Beagle 12
Beagle 13
Beagle 20
Beagle 24
IFAT
ELISAp
ELISAsla
CS
Estimation MW (KDa)
>97
97
93
87
85
77
72
70
66
56
50
48
46
44
41.5
40
38
35
32
30
28
25.5
23.5
23
21.5
19.5
17
15
Total DU
203.8
905.7
191.5
3,288.5
141.9
0.0
5,933.7
233.2

SI Figure 3A

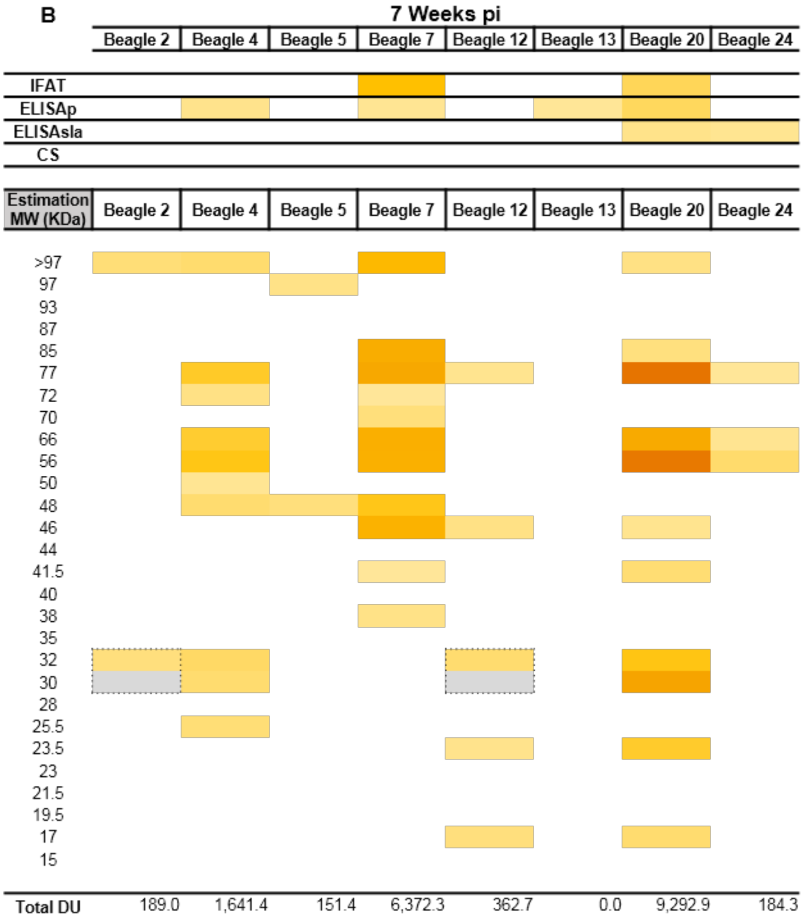
B
7 Weeks pi
Beagle 2
Beagle 4
Beagle 5
Beagle 7
Beagle 12
Beagle 13
Beagle 20
Beagle 24
IFAT
ELISAp
ELISAsla
CS
Estimation MW (KDa)
>97
97
93
87
85
77
72
70
66
56
50
48
46
44
41.5
40
38
35
32
30
28
25.5
23.5
23
21.5
19.5
17
15
Total DU
189.0
1,641.4
151.4
6,372.3
362.7
0.0
9,292.9
184.3

SI Figure 3B

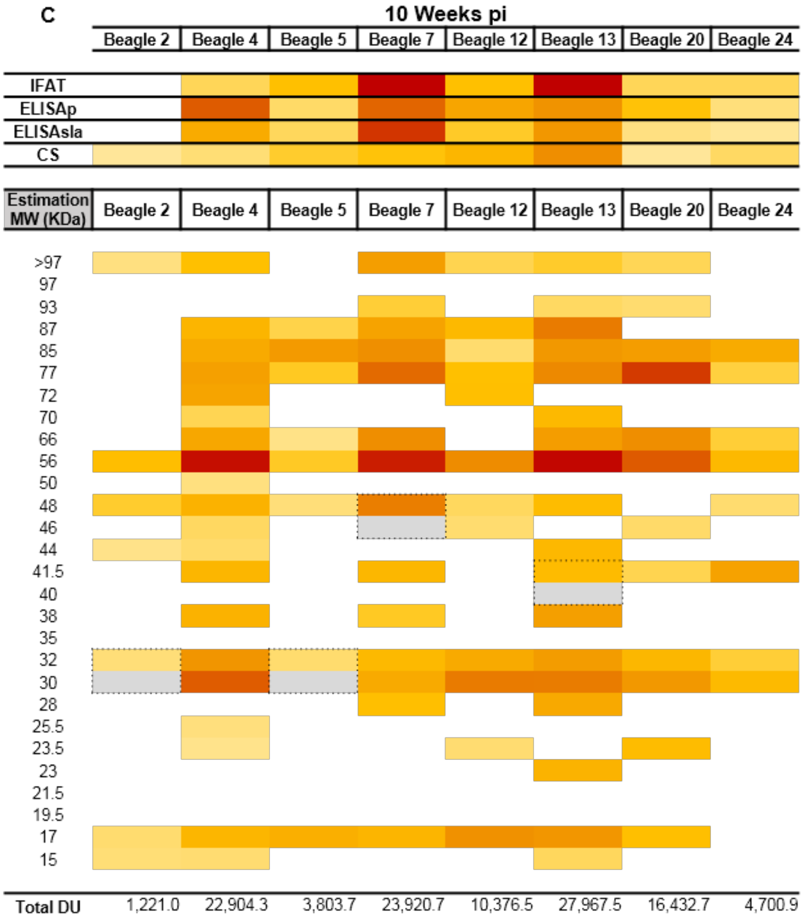
C
10 Weeks pi
Beagle 2
Beagle 4
Beagle 5
Beagle 7
Beagle 12
Beagle 13
Beagle 20
Beagle 24
IFAT
ELISAp
ELISAsla
CS
Estimation MW (KDa)
>97
97
93
87
85
77
72
70
66
56
50
48
46
44
41.5
40
38
35
32
30
28
25.5
23.5
23
21.5
19.5
17
15
Total DU
1,221.0
22,904.3
3,803.7
23,920.7
10,376.5
27,967.5
16,432.7
4,700.9

SI Figure 3C

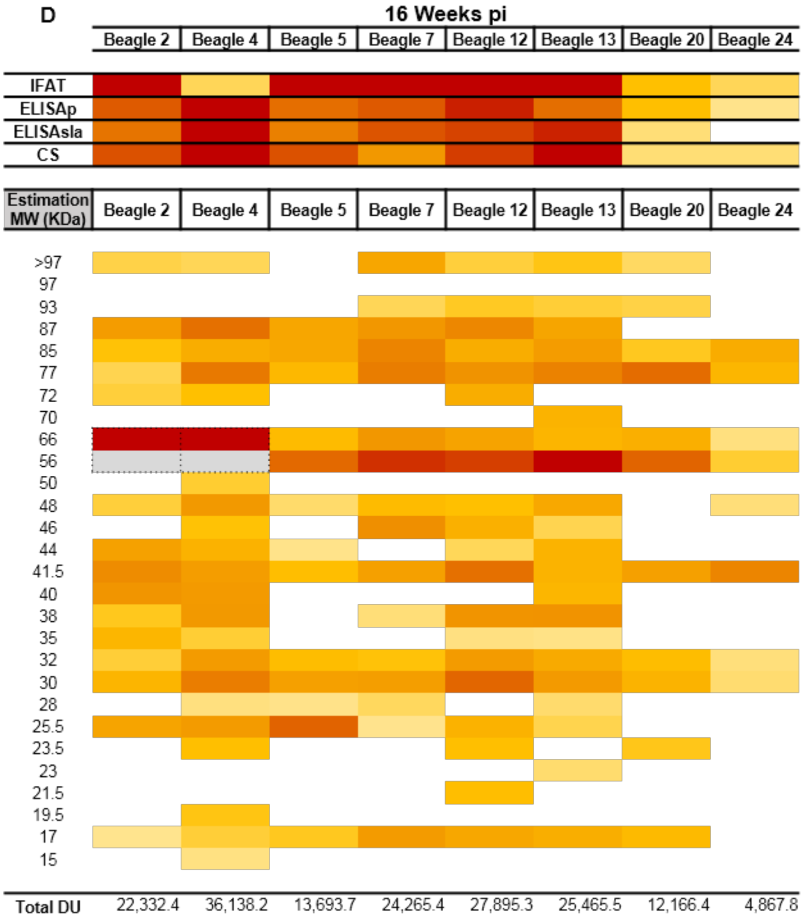
D
16 Weeks pi
Beagle 2
Beagle 4
Beagle 5
Beagle 7
Beagle 12
Beagle 13
Beagle 20
Beagle 24
IFAT
ELISAp
ELISAsla
CS
Estimation MW (KDa)
Beagle 2
Beagle 4
Beagle 5
Beagle 7
Beagle 12
Beagle 13
Beagle 20
Beagle 24
>97
97
93
87
85
77
72
70
66
56
50
48
46
44
41.5
40
38
35
32
30
28
25.5
23.5
23
21.5
19.5
17
15
Total DU
22,332.4
36,138.2
13,693.7
24,265.4
27,895.3
25,465.5
12,166.4
4,867.8

SI Figure 3D

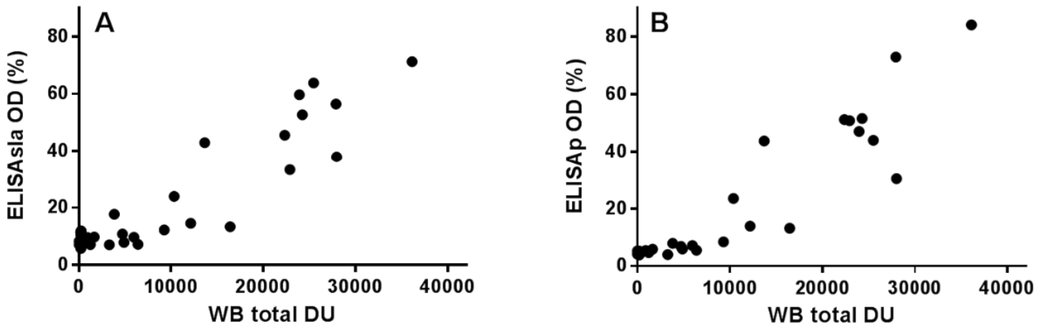
A
ELISAsla OD (%)
WB total DU
B
ELISAp OD (%)
WB total DU
0
20
40
60
80
0
10000
20000
30000
40000

SI Figure 4

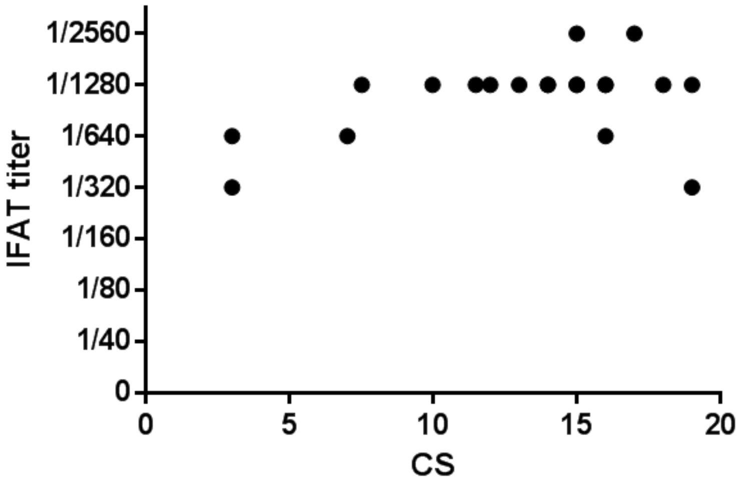

SI Figure 5

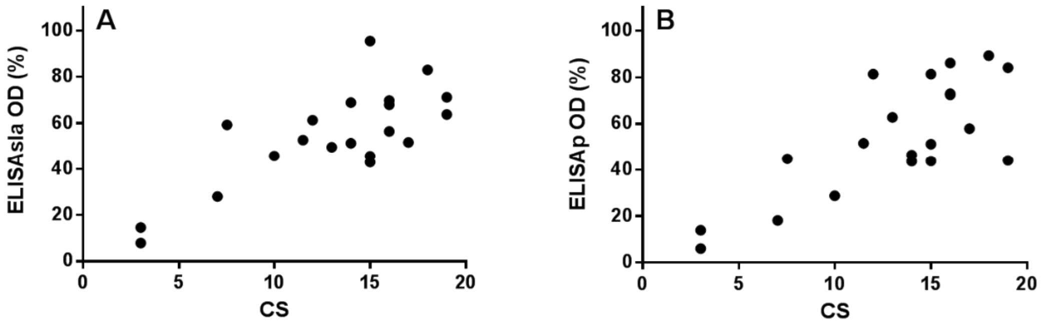

SI Figure 6

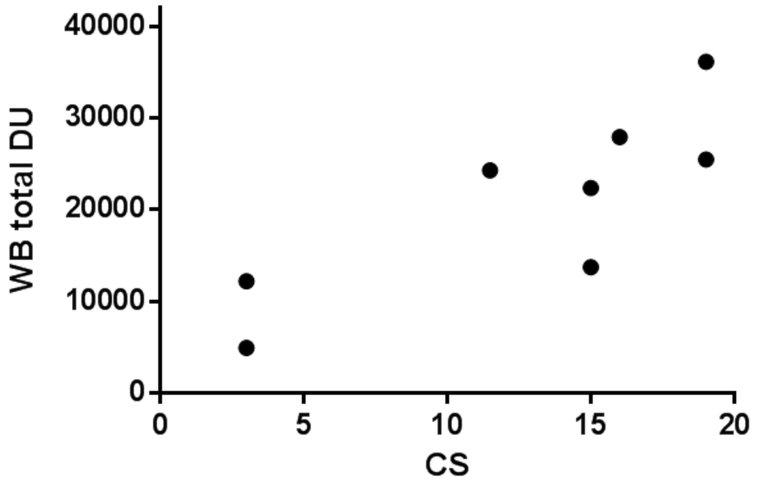

SI Figure 7

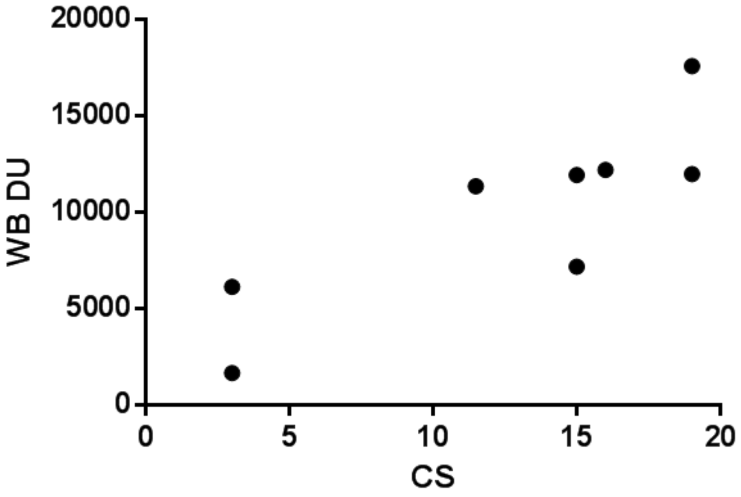

SI Figure 8

SI Table 1: Alignment of identified peptides by Mass Spectrometry and finger printing in the 2D-electrophoresis (in bold) and the sequences of proteins of *Leishmania* or trypanosomatids.

Protein nº	Protein identification	Matched peptides
1	Heat shock protein 83-1 (HSP 83)	**1** SNKEIFLREL ISNASDACDK IR**YQSLTDPS VLGDETR**LRI RVIPDKANKT<br>**51** LTVEDNGIGM TK**ADLVNNLG TIAR**SGTKAF MEALEAGGDM SMIGQFGVGF<br>**101** YSAYLVADRV TVVSKHNADE AYVWESSAGG TFTIATVADS DLKRGTRITL<br>**151** HLK**EDQQEYL EER**RVKELIK K**HSEFIGYDI ELLVEKTTEK** EVTDEDEEEK<br>**201** KEGXNEEEPK VEEVKEGEED KKKTKKVKEV TKEYEIQNK**H KPLWTR**DPK**D**<br>**251** **VTKEEYAAFY K**AISNDWEDP AATK**HFSVEG QLEFR**SILFV PK**RAPFDMFE**<br>**301** **PNK**KRNNIKL YVRRVFIMDN CEDLCPDWLG FVK**GVVDSED LPLNISR**ENL<br>**351** QQNKILKVIR KNIVKKCLDL FEELSENKED FKQFYEQFGK NLK**LGIHEDT**<br>**401** **ANR**K**KLMELL RFASTESGEE LTTLK**DYVTR MKPEQK**SIYY ITGDSK**K**KLE**<br>**451** **SSPFIEEAKR** RGIEVLFMTE PIDEYVMQQV KDFEDKKFAC LTKEGVHFEE<br>**501** SEDEKKKREE DKTAYEKLCK AMKEILGDKV EKVAVSERLS TSPCILVTSE<br>**551** FGWSAHMEQI MRNQALRDSS MAQYMMSKKT MELNPGHPII RELRRR**VEAD**<br>**601** **ENDKAVK**DLV FLLFDTSLLT SGFQLDDPTG YAERINR
2	Putative methylmalonyl-coenzyme A mutase	**1** MMRRCCILLA DAAAAGGGYP PEWIATAKKE LKRDPSTLVR HSINGFDIKP<br>**51** LYLPEDVKKV PNLPGFFPFT R**GVHATMYTG RPWTIRQYAG FSTAEESNNF**<br>**101** **YK**AALKSGQQ GLSVAFDLAT HR**GYDSDHPR** VTGDVGMAGV AVDTVEDMKL<br>**151** LFKDIPLDK**V SVSMTMNGGV IPILAFFAVA AEESGVPQAK** LRGTIQNDIL<br>**201** KEFMVRNTYI FPPTPSMRII GDIMAYLNKN QPKFNSISIS GYHIQEAGAD<br>**251** GALELAFTIA DGLEYIRCAE AR**GLTVDDVA PRLSFFFGIG MNFYCEIAK**L<br>**301** RAARTLWATY VKKKFNPKNS KSLLLR**THSQ TSGWSLTEQD MQNNIIR**TTI<br>**351** EAMAAVMGGV QSLHTNAFDE AVALPSKQSS RTARNTQIII QEETHICGVV<br>**401** DPWGGSYMME ALTQEMIDR**A TAIIEDVESK** GGMTKCIEEG FPKLKVEESA<br>**451** AR**RQAAIDSG AETIVGVNK**Y VNPDDKAPET LRIDNEKVRA GQIAGIQRVK<br>**501** AARDTAKCEA ALKQVTAACK DNSINILDVA IVAARERATL GEITFAMEEV<br>**551** YGRYVAKNQV VQGVYYSTYV KDGSKEEQDY VGKIKARIDA YAAKEGRRPR

		**601** **IMVAKMGQDG HDR**GAKVVAT GLADMGYDVD IGPLFQTPEE VARHAVENDV<br>**651** HIVGASSLAA GHRTLIPQLI QELKKLGADD IIVTAGGVIP PGDYQELYDA<br>**701** GVKMIFGPGT PIPKCADEMI TALEARQK
3	Putative heat-shock protein hsp70 (Fragment)	**1** MTFEGAIGID LGTTYSCVGV WQNERVDIIA NDQGNRTTPS YVAFTDSERL<br>**51** IGDAAK**NQVA MNPHNTVFDA KR**LIGRKFND SVVQSDMKHW PFKVTTKGDD<br>**101** KPVIAVQYRG EEKTFTPEEI SSMVLLKMKE TAEAYLGKQV KK**AVVTVPAY**<br>**151** **FNDSQR**QATK **DAGTIAGLEV LR**IINEPTAA AIAYGLDKGD DGKERNVLIF<br>**201** DLGGGTFDVT LLTIDGGIFE VKATNGDTHL GGEDFDNRLV TFFTEEFKRK<br>**251** NKGKNLASSH RALRRLRTAC ERAKRTLSSA TQATIEIDAL FENVDFQATI<br>**301** TR**ARFEELCG DLFR**STIQPV ERVLQDAKMD KRSVHDVVLV GGSTRIPKVQ<br>**351** SLVSDFFGGK ELNKSINPDE AVAYGAAVQA FILTGGKSKQ TEGLLLLDVT<br>**401** PLTLGIETAG GVMTALIKRN TTIPTKK**SQI FSTYADNQPG VHIQVFEGER**<br>**451** **AMTKDCHLLG TFDLSGIPPA PRGLPQIEVT FDLDANGILN VSAEEK**GTGK<br>**501** R**NQITITNDK** GRLSKDEIER MVNDAMKYEE DDKAQRDRVE AKNGLENYAY<br>**551** SMKNTLSDSN VSGKLEDSDK ATLNKEIDVV LEWLSSNQEA AKEEYEHKQK<br>**601** ELESVCNPIM TK**MYQSMGGA GGGMPGGMPD MSGMSG**
4	Chaperonin HSP60, mitochondrial precursor	**1** MFRSAVRFGA K**DIRFGTEAR** QSMLKGVQRA VDAVATTLGP KGRNVIIEQS<br>**51** YGAPKITKDG VTVAKSIEFK DPFENMGAQL VRQVCNKTND LAGDGTTTSA<br>**101** VLVASIFSEG IK**CIATGTNP IDMK**R**GMDRA VDVILK**SIES QSRKVTSTEN<br>**151** VVQVATISAN GDVELGKLIG EAMEKVGK**DG VITTQDGK**TL TTELEVVEGM<br>**201** SIDR**GYISPY FVTDAK**TQKA ELEEAFVLVS AKKLSNIHTI LPALNHVVRS<br>**251** GRPLLIIADD VESEALTTMI FNKLQGKLKI ACVKAPGFGD NKAATLQDIA<br>**301** IFSGARVVGE EGSGVELDAD NFDPDILGSV KKATITKDDT VLLNGGGDSG<br>**351** LVKERVELLR GLIENETSDY NREKLQERLG K**LSGGVAVIR** VGGASEVEVN<br>**401** EKK**DRITDAL CSTRAAVQEG IVPGGGAALL RASK**ELEGLL NDQSLTADQR<br>**451** TGVQIIRNAV RLPAHRIVSN SGREGAVVVE KVLENGDK**AV GYDAQLDR**YV<br>**501** NMFDAGIIDP ARVVRVALTD AASVASLMMT AEAAVVDLPK EDPPAAGGMG<br>**551** GMGGMGGMGG MGGMY
5	Elongation factor 1-alpha	**1** MGKASFK**YAW VLDKLK**AERE R**GITIDIALW K**FESPK**SVFT IIDAPGHR**DF<br>**51** IK**NMITGTSQ ADAAILMIDS TQGGFEAGIS K**DGQTREHAL LAFTLGVKQM<br>**101** VVCCNKMDDK TVQYSQARYE EISK**EVGTYL KR**VGYNPEKV RFIPISGWQG<br>**151** DNMIDKSESM AWYK**GPTLLD ALDMLEAPVR PVDKPLRLPL QDVYKIGGIG**<br>**201** **TVPVGR**VETG IMKPGDVVTF APANVTTEVK **SIEMHHEQLA EAVPGDNVGF**<br>**251** **NVK**NVSVKDI RRGNVCGNSK NDPPKEAADF TAQVIVLNHP GQISNGYAPV

		**301** LDCHTSHIAC RFADIESKID RRSGKELEKN PKAIKSGDAA IVK**MVPQKPM**<br>**351** **CVEVFNDYPP LGR**FAVRDMR **QTVAVGIIK**A VSKK**DGSAGK VTK**AAAKAAK<br>**401** K
6	Enolase	**1** MTIQKVHARE ILDSRGNPTV EVEVTTDKGV FRSAVPSGAS TGVHEACEMR<br>**51** DEDKGRYCGK GCLKAVKNVN EVLGPALVGK DETQQEVLDK LMCDLDGTKN<br>**101** K**SKLGANAIL GCSMAISK**AA AARLGLPLYQ YIAKIAGTKE IRLPVPCFNV<br>**151** INGGK**HAGNV LPFQEFMIAP VK**AKSFREGL QMGAEVYHAL KSILKKKYGQ<br>**201** DAVNVGDEGG FAPPIAHIDE PLPILMEAIE KAGHKDRFAI CMDCAASEAY<br>**251** DADKKQYNMT FKSAEPTYVS GEGLLKTYEK WATNYPIKSI EDPFSEDNFD<br>**301** EFAAITKALE GK**VQIVGDDL TVTNVER**VKM AIEKKACNSL LLKVNQIGTV<br>**351** SESIAAAR**LC MDNGWSVMVS HR**SGETEDTY IADLSVGLGT GQIKTGAPCR<br>**401** SER**TAKMNQL LR**IEEELGAS SKYGFPAWA
7	Putative heat shock protein DNAJ	**1** MVAETK**YYDA LGVSPTATED EIKR**AYRKLA LKYHPDKNKE PGAQEKFK**EV**<br>**51** **SVAYECLSDP EKR**KLYDQFG DKGEGMESGI DPSDIFASFF GGGTRSRGEP<br>**101** KPKDIIHELP VSLDAFYTGK TVKLAITRDR LCTKCSGTGS KIPNASIKCR<br>**151** ECNGRGVRMI TRQIGPGFIQ QMQVTCPACQ GKGTSLKEED KCEVCRGQQT<br>**201** IKDKKIFEVV VDKGMHRGDS VTFRGEGDQI PDVRLSGDII IIFEQKPHPT<br>**251** FIRKGNHLFI ERSISLAEAL TGFSFNITHL DNRKLK**IQSP EGMVVDPANM**<br>**301** **YSVHR**EGMPV PNTGGVEK**GD LVIKFNVVFP K**KMEQSLIPN LRSTLGYPHQ<br>**351** PKSDHDSEMC ILQETKIDLE KESRRNAYDD DGDDNRPRGH TTTCAQQ
8	Arginine kinase (AK)	**1** MASPDVIAKL DAAFSK**LQNA SDCNSLLK**K**H LTKNVFEEIK** GRKTKLGATL<br>**51** LDVIQSGVAN LDSGVGLYAP DAESYTVFAP LFDPVIEDYH KGFKPSDRQP<br>**101** PKDFGDLSTL VDVDPDNKYV LSTRVRCGRS LEGYPFNPCL TKAQYEEMES<br>**151** RVK**EQLSTMT GELQGCYYPL TGMTK**ETQQK LIDDHFLFKE GDRFLQAARA<br>**201** CEHWPTGRGI YHNENK**TFLV WVNEEDHLR**I ISMQKGGNLK EVFGRLVTAV<br>**251** GIIEQKVKFS RDDR**LGFLTF CPTNLGTTIR** ASVHIKLPKL GADRAKLEEV<br>**301** AAKYSLQVRG TAGEHSDSPD GVYDISNKR**R LGLSEYQAVK** EMQDGILELI<br>**351** KIEQSLDGNQ DDNALHNFFR SLSKI
9	Putative glutathione peroxidase-like protein	**1** MSIYDFKVNG GDHKPYDLGQ HK**GHPVLIYN VASK**CGFTK**G GYETATALYN**<br>**51** **K**YKHQGFTVL AFPCNQFASQ EPGTEESVKE FACTRFKAEF PIMEKVCVNG<br>**101** EHEHPLYHYL K**NTCKGVLGT TLVK**WNFTAF LVDKDGHAVC RFAPGATMSE<br>**151** IEKRLVPLLE ADGDASTAPL STQA

Supporting Information Table 2: Clinical scoring (CS) of Beagle dogs experimentally infected with *Leishmania infantum* considering clinical signs and lesions (20 points) and hematological and biochemical abnormalities (15 points). Maximum Score: 35 points

**Clinical signs & Lesions**					
		**Severity score**			
		**0**	**1**	**2**	**3**
Lymphadenomegaly	Enlargement of palpable lymph nodes (LN)<br>▪ Popliteal LN<br>▪ Retropharyngeal LN<br>▪ Prescapular LN	0 (Normal)	1 (1 palpable LN, localized lymph.)	2 (> 1 palpable LN, mild lymph.)	3 (generalized, severe lymph.)
Splenomegaly	Spleen enlargement as determined by palpation	0 (absence)	1 (slight)	2 (moderate)	3 (severe)
Cutaneous lesions	▪ Non-pruritic dry exfoliative dermatitis<br>▪ Alopecia (periocular/ focal/ generalized)<br>▪ Erythema<br>▪ Ulcerative dermatitis<br>▪ Nodular dermatitis<br>▪ Papular dermatitis<br>▪ Pustular dermatitis<br>▪ Intertrigo<br>▪ Onychogryphosis<br>▪ Nasal and/or footpad hyperkeratosis<br>▪ Pyoderma	0 (Normal)	1 (localized or mild lesions and/or alopecia)	2 (multifocal or generalized lesions and/or severe alopecia)	-
Weight loss	▪ % decrease of body weight	0 (absence)	1 (slight < 5%)	2 (moderate 5-10 %)	3 (severe ≥ 10 %)
Body condition loss	As classified by the veterinarian	0 (Normal)	1 (poor) condition)	2 (emaciation)	3 (cachexia)
Mucosal pallor	▪ Oral mucosa<br>▪ Vulvar mucosa	0 (pink and moist)	1 (pale mucosa)	-	-
Hyperthermia	▪ Fever (> 39°C)	0 (absence)	1 (presence)	-	-
Ocular lesions	▪ Blepharitis (exfoliative, ulcerative, or nodular) and conjunctivitis (nodular)<br>▪ Keratoconjunctivitis, either common or sicca<br>▪ Anterior uveitis/endophthalmitis	0 (absence)	1 (presence)	-	-
Gastrointestinal signs	▪ Diarrhea<br>▪ Vomiting	0 (absence)	1 (presence)	-	-
Muscular Atrophy	▪ Masticatory muscle atrophy (temporal muscle)	0 (absence)	1 (presence)	-	-
Joints	▪ Lameness<br>▪ Joint pain	0 (absence)	1 (presence)	-	-
				**TOTAL SCORE**	**20**

**Hematological and Biochemical abnormalities**					
Anemia	▪ Hematocrit value (<38.3%)<br>▪ Reticulocytes cell count (<110000 cells/µL): non regenerative anemia	0 (absence)	1 (mild to moderate anemia)	2 (mild to moderate non regenerative anemia)	-
Thrombocytopenia	▪ Platelets (<143000/µL)	0 (absence)	1 (presence)	-	-
Leucopenia	▪ Leucocytes (<4900/µL)	0 (absence)	1 (presence)	-	-
Neutrophilia	▪ Band neutrophils (>170 cells/µL)	0 (absence)	1 (presence)	-	-
Increased total proteins	▪ Proteins spectrophotometry (> 7.8 g/dL)	0 (absence)	1 (presence)	-	-
Globulinemia	▪ Globulins (> 4.4 g/dL)	0 (absence)	1 (presence)	-	-
Hypoalbuminemia	▪ Albumin (< 2.7 g/dL)	0 (absence)	1 (presence)	-	-
Decreased albumin/globulin ratio	▪ Ratio A/G < 0.7	0 (normal: A/G = 0.7-1.9)	1 (A/G= 0.7-0.35)	2 (A/G < 0.35)	
Renal impairment or renal disease	▪ Blood creatinine concentration (> 1.5 mg/dL)	0 (normal)	1 (presence)		
	▪ Blood Urea Nitrogen (> 59 mg/dL)	0 (absence)	1 (presence)		
	▪ SDMA (symmetric dimethylamine) biomarker of renal function (> 14 µg/dL)	0 (absence)	1 (presence)		
Increased liver enzymes	GOT (AST) (> 89 UI/dL)	0 (absence)	1 (presence)		
	GPT (ALT) (> 89 UI/dL)	0 (absence)	1 (presence)		
				**TOTAL SCORE**	**15**
